# Supplementary material for: Structural changes in endocrine pancreas of male Wistar rats due to chronic cola drink consumption. Role of PDX-1
Source: PLoS One. 2021 Jun 11;16(6):e0243340. doi: 10.1371/journal.pone.0243340 (PMC8195359; doi:10.1371/journal.pone.0243340)
Supplement: Attachment — Submitted filename: PONE-D-20-36227_reviewer coments.pdf [file pone.0243340.s005.pdf]

PLOS ONE

Manuscript No. PONE-D-20-36227 10th January 2021

Dear Editor Muhammad Sajid Hamid Akash, PhD

TITLE: Could TDX-1 explain the changes observed in endocrine pancreas due to chronic cola drink consumption in rats? A global point of view

Author: Gabriel Cao.

Comments to the Author

This manuscript nice presented and interesting finding to assess the impact of the three different beverages in glucose tolerance, lipid levels, creatinine levels, and immunohistochemical changes addressed for the expression of insulin, glucagon, PDX-1 and NGN3 in islet cells, to evaluate the possible participation of PDX-1 in the changes observed in α and β cells after 6 months of treatment. Overall, these findings are important and interesting. However, further, improvement is necessary to solidify the Manuscript.

Here are a few comments and questions:

1. In the current manuscript, the author used the title Could "TDX-1 explain the changes observed in the endocrine pancreas due to chronic cola drink consumption in rats? A global point of view. Revised the title. The title does not completely match with the study.
2. To solidify the idea of the PDX-1 current study, the author should add more experiments like real-time PCR result or western blot results.
3. There is some typing error in table:2
4. In the current study what the effect of chronic or light cola on liver function test after 6 months. Like ALT, AST, ALP Bilirubin, etc.
5. Confusion in the group of chronic cola and light cola was only given cola for drinking.?

Authors address these deficiencies, then the manuscript should be considered for publication.
